# Relationship between danofloxacin PK/PD parameters and emergence and mechanism of resistance of *Mycoplasma gallisepticum* in *In Vitro* model

**DOI:** 10.1371/journal.pone.0202070

**Published:** 2018-08-29

**Authors:** Nan Zhang, Yuzhi Wu, Zilong Huang, Chuanzhen Zhang, Longfei Zhang, Qinren Cai, Xiangguang Shen, Hongxia Jiang, Huanzhong Ding

**Affiliations:** 1 School of life science and engineering, Foshan University, Foshan, China; 2 Guangdong Key Laboratory for Veterinary Drug Development and Safety evaluation, South China Agricultural University, Guangzhou, China; 3 Technical Center for Inspection & Quarantine, Zhuhai Entry-Exit Inspection and Quarantine Bureau, Zhuhai, China; The University of Melbourne, AUSTRALIA

## Abstract

*Mycoplasma gallisepticum* is a serious pathogen for poultry that causes chronic respiratory disease in chickens. Increased embryonic mortality, as well as reduced weight gain and egg production have been found in infected chickens, which can lead to considerable economic losses in poultry production. Increased antibiotic resistance compromises the use of tetracyclines, macrolides and quinolones in the farm environment. In the present study, danofloxacin concentrations were simulated below the MIC_99_, between the MIC_99_ and MPC (the mutant prevention concentration), and above the MPC in an *in vitro* dynamic model against *M*. *gallisepticum*. The relationship between the simulated danofloxacin pharmacokinetics, pharmacodynamics (PK/PD) parameters and development of resistance for *M*. *gallisepticum* was explored based on the available data obtained from various dosing regimens in the *in vitro* model. Danofloxacin concentration, counts of viable cell and susceptibility were determined during the experiment. The mutations in *gyrA*, *gyrB*, *parC* and *parE* as well as efflux pumps were examined. The MIC of danofloxacin against *M*. *gallisepticum* was increased when drug concentrations were between the lower and upper boundaries of the mutant selection window. The upper boundary of the selection window *in vitro* was estimated as a C_max_/MPC value of 1. The lower boundary was estimated as C_max_/MPC value of 0.05. Both in terms of the MIC and resistance frequency, *M*. *gallisepticum* resistance was developed when danofloxacin concentrations fell inside the mutant selection window (ratios of C_max_ to MPC between 0.05 and 1). The single mutation in *gyrA* (Ser-83→Arg) was found in all mutants, while double mutations in *gyrA* and *parC* (Ala-64→Ser) were observed only in the mutant with the highest MIC. In addition, no change of susceptibility in the mutants was observed in the presence of reserpine and carbonyl cyanide 3-chlorophenylhydrazone (CCCP). This suggested that ATP-binding cassette superfamily (ABC transporter) and major facilitator superfamily (MFS transporter) did not play a role in danofloxacin efflux.

## Introduction

*Mycoplasma gallisepticum* is a major pathogen causing chronic respiratory infections in poultry [1]. Infected birds exhibit mostly respiratory signs including ocular and nasal discharge, rales and sneezing. In some instances, infected flocks have high mortality rates. Mild cases are reflected in reduced egg production and feed conversion rates [2]. *M*. *gallisepticum* transmission throughout avian populations occurs by both horizontal and vertical routes. Even though numerous control measures are in place to eradicate this disease, a heavy reliance on antibiotics has become the *de facto* control strategy. The quinolones, macrolides, tetracyclines and pleuromutilin are all effective against *M*. *gallisepticum* [3, 4]. Danofloxacin, is now widely used in China for controlling the respiratory disease from *M*. *gallisepticum* in chickens.

The “mutant selection window” (MSW) hypothesis is a concept devised by Zhao and Drlica [5], which postulates resistant mutants selectively and amplify at antibiotic concentrations within the mutant selection window (MSW). The lower boundary of the range is the MIC_99_ (inhibition of 99% of the cells) that serves as a threshold for restricting the growth of the majority of susceptible bacteria. The mutant prevention concentration (MPC) that inhibits growth of the least susceptible single-step mutant subpopulation is defined as the upper boundary [6]. The MSW hypothesis has been tested in several *in vitro* and *in vivo* models [7–12].

*M*. *gallisepticum* is minute in size and completely lacks a bacterial cell wall. It can only be cultivated on specially formulated media due to its dependence on external sources of precursor molecules for macromolecular syntheses [13, 14]. Moreover, quantitative cultures by viable count estimation (CFU determination) are complicated owing to these strict nutritional conditions and the long growth cycle (7 days). Hence, there are few reports concerning PK/PD integration of antimicrobials against *M*. *gallisepticum* and its drug resistance mechanisms. In the current study, we simulated different danofloxacin dosing regimens using an *in vitro* dynamic model to obtain the related PK/PD parameters. Our objective was to investigate the relationship between the danofloxacin PK/PD indices and mutant selection enrichment of *M*. *gallisepticum*. The *gyrA*, *gyrB*, *parC* and *parE* genes in danofloxacin resistant strains were sequenced to identify mutations in the quinolone resistance-determining region (QRDR). Furthermore, the active danofloxacin efflux that has never been evaluated in *M*. *gallisepticum* was also examined. It was expected that our investigation would illustrate the relationship between dosing regimens and mutant selection enrichment, and more clearly elucidate genetic mutations associated with danofloxacin-resistant subpopulations of *M*. *gallisepticum*.

## Materials and methods

Danofloxacin powders were provided by Guangdong Wens Dahuanong Biotechnology Co.Ltd. (Xinxing, China). *M*. *gallisepticum* strain S6 used in this study was supplied by the Chinese Veterinary Microorganism Culture Collection Center (Beijing, China). The components used for PCR (polymerase chain reaction) were obtained from Takara Bio (Ohtsu, Japan).

### MIC_99_, MIC, and MPC Determinations

The minimum inhibitory concentration (MIC) of danofloxacin was determined by the agar dilution method as described elsewhere [15]. Briefly, 10 μL samples of *M*. *gallisepticum* culture (10^7^ CFU/mL) were inoculated onto agar plates containing two-fold serial dilutions of danofloxacin to determine the minimal drug concentration that resulted in no growth in 7 days.

MIC_99_ and MPC measurements were performed following the previously described method with modifications [16]. For MIC_99_, 10^7^ CFU/mL log phase cells were diluted in a 10-fold series and 10 μL of each diluted suspension were applied to agar plates containing linearly decreasing danofloxacin concentrations. The two modifications of the measurement of MIC_99_ were as following: Firstly, these concentrations were based on the MIC value and decreased 10% per step in a range of 1 × MIC to 0.5 × MIC instead of 20% steps. Obviously, the 10% per sequential decrease was used to improve the accuracy of MIC_99_ determination, which was more complicated than ever done in the past. Secondly, the inoculum size of *M*. *gallisepticum* was 10 uL. This parts have been explained in more detail in the other article [17]. Colony numbers were determined using an inverted microscope after 7-days at 37°C in a 5% CO_2_ humidified atmosphere. Drug concentrations versus colony recoveries were plotted and MIC_99_ was calculated by interpolation.

For MPC, more than 10^10^ CFU were spread on agar plates containing a series of danofloxacin concentrations. Provisional mutant prevention concentration (MPCpr) was defined as the lowest antimicrobial concentration that prevented colony formation. These cells were then cultivated on plates that utilized linear danofloxacin concentration decrements (about 20% per sequential decrease). The MPC was recorded as the lowest drug concentration preventing 100% growth.

### *In vitro* dynamic model and the pharmacokinetic profiles

**Fig 1:**
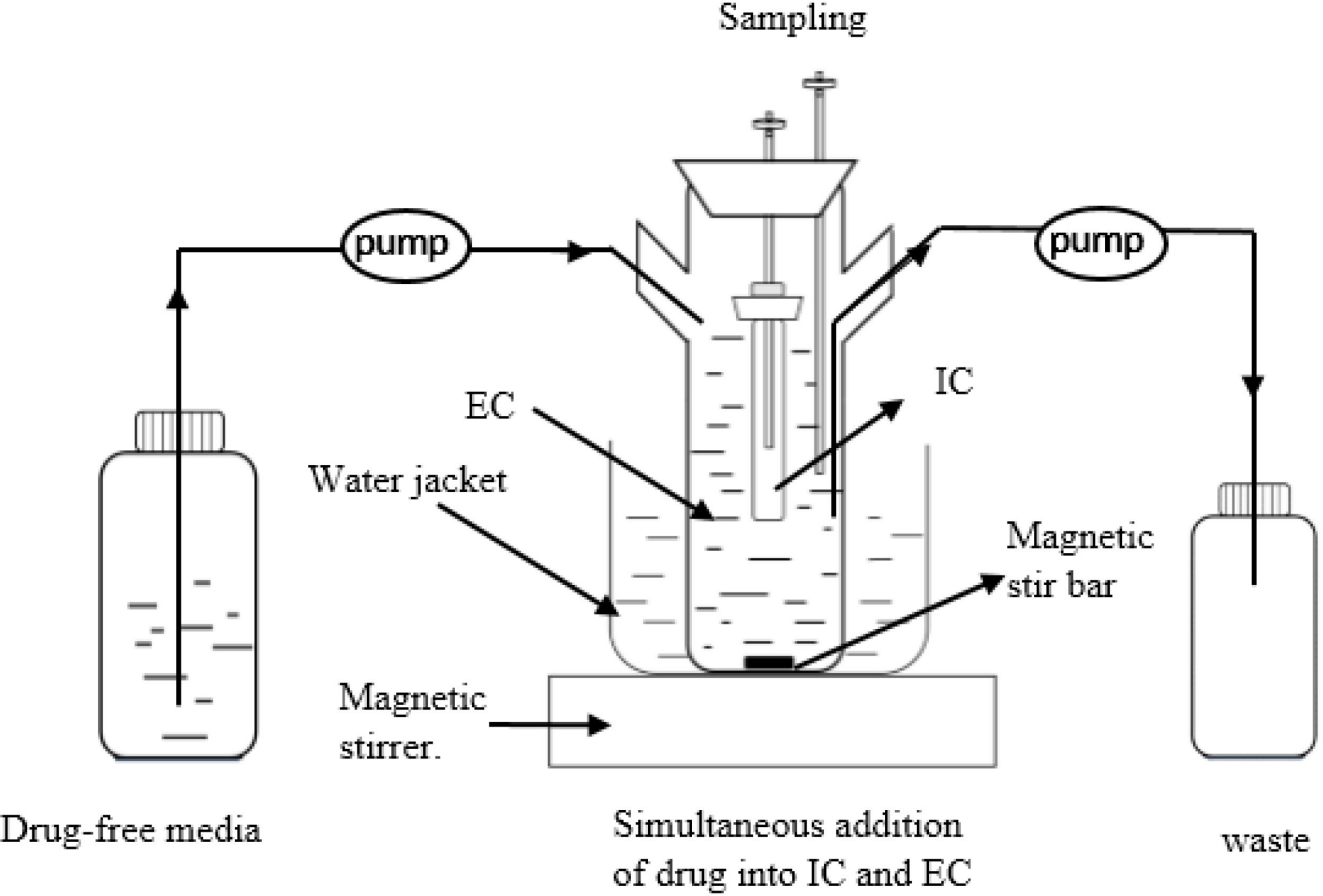
The *in vitro* model that simulates the pharmacokinetics of danofloxacin in lung tissues of the *M*. *gallisepticum* infected chickens and determines a drug’s effect on growth and susceptibility of *M*. *gallisepticum*.

The *in vitro* dynamic model has been described previously [18, 19]. The *in vitro* PK/PD simulation model used in this study is shown in Fig 1. Briefly, the model is connected by three glass flasks. The first one containing fresh medium without antimicrobials; the second is the external compartment [EC] that consists of a three-necked flask containing 320 mL medium and a cellulose dialysis tube whose 10 mL interior volume is defined as the internal compartment [IC]; the third is the waste flask. The IC is made of cellulose membrane that restricts the passage of large molecules. It contains medium and inoculum, while the EC contains only the medium. The EC is a three-necked flask maintained at 37°C with constant stirring. The drug-free medium is pumped continuously into the EC from the reservoir to dilute the drug, and a second pump ensuring the discharging.

For all experiments, 10 mL of culture containing 10^9^ CFU/mL of *M*. *gallisepticum* was inoculated into the IC as the starting inoculum. At time zero, danofloxacin was added to both compartments in order to allow rapid equilibrium. The danofloxacin concentrations in lung tissues of the *M*. *gallisepticum* infected chickens were simulated using this *in vitro* model. A series of monoexponential profiles that mimic once-daily administration of danofloxacin was simulated for 5 consecutive days. The simulated half-life of danofloxacin of 10 h was consistent with that reported in chickens [20–22]. With danofloxacin-exposed cells, the simulated C_0_ in the *in vitro* model were 0.1, 0.3, 0.675, 1.25, 2, 7, and 13 mg/L (C_0_ was the initial danofloxacin concentration). After each administration, 1 mL samples were collected at 1, 3, 6, 9, 12, and 24 h from EC to determine danofloxacin concentration. Additionally, the samples from the IC were also collected to generate a time-killing curve and monitor the potential loss of danofloxacin susceptibility.

### Determination of danofloxacin concentration

EC samples were stored at -20°C until they were assayed. The concentrations of danofloxacin were analyzed using high performance liquid chromatography with fluorescence detection (HPLC-FD) (Agilent Technologies, USA). The sample (1 ml) was added to 0.5 mL acetonitrile, vortexed for 1 minute and incubated in a 45°C water bath for 10 min to precipitate proteins. The samples were centrifuged at 10, 000 × g for 5 min at 4°C. Then 0.5mL supernatant was transferred to a centrifuge tube containing 0.5 mL ultra-pure water. Finally, the mixture was vortexed for 30 s and filtered through a 0.22 μm syringe filter prior to HPLC analysis. An Agilent TC-C18 column (250 mm × 4.6 mm, 5 μm) was was used for isocratic separation with a mobile phase of trimethylamine phosphate (pH 2.4): acetonitrile (19:81, v/v) at 0.8 mL/min. A calibration curve was established in triplicate with seven danofloxacin concentrations (0.001–0.1 μg/mL).

PK/PD indices such as AUC_24_/MIC_99_ (the area under the concentration-time curve over 24 h divided by the MIC_99_), AUC_24_/MPC (the area under the concentration-time curve over 24 h divided by the MPC), C_max_/MIC_99_ (the peak concentration divided by the MIC_99_), and C_max_/MPC (the peak concentration divided by the MPC) were calculated using the WinNonlin program (version 6.1, Pharsight Corporation, Mountain View, CA, USA).

### The time-killing study and susceptibility changes

In each experiment, samples were obtained daily before and during the danofloxacin treatment (at 24 h after every administration and at 48 h after the termination of treatment). The time-killing study and the potential loss of susceptibility were monitored throughout the observation period. The potential loss of susceptibility was measured using the MIC determination and the fraction of surviving mutants. In brief, half of each sample was incubated in drug-free growth medium and the MIC was determined according to the agar dilution method as mentioned above. The other part of the collected sample was diluted serially in growth medium and spotted on agar plates that were either free of drug or contained danofloxacin at 1 × MIC. After incubation in a 5% CO_2_ humidified incubator at 37°C for 7 days, colonies were counted to determine the fraction of mutants in the population. The resistant mutants were also selected randomly. After susceptibility determination, the mutants displaying increased MICs to danofloxacin were screened for QRDR mutations in of *gyrA*, *gyrB*, *parC*, and *parE*. In addition, the selected single colonies were passaged for 5 times. The isolates were then subjected to danofloxacin susceptibility testing and screened for QRDR mutations to detect whether the mutations could be inherited steadily.

### Resistance analysis

Mutants in which the MIC increased to 0.3, 1.2, 2.4 and 4.8 mg/L using the agar diffusion method were selected for DNA extraction using a previously described protocol [23]. The mutant colonies were randomly selected from the agar plates, and three mutant strains at each MIC were selected to study the resistance mechanisms. The QRDR gens *gyrA*, *gyrB*, *parC* and *parE* were amplified by the specific primers designed from the strain S6 of *M*. *gallisepticum* [24]. Amplified reaction was achieved as described previously with modification [25]. PCR mixture was consisted of 2.5 μL 10 × Ex Taq buffer (Mg^2+^ plus), 2.5 μL dNTP, 0.5 μL primer F (10 μmol/mL), 0.5 μL primer R (10 μmol/mL), 1 μL extracted *M*. *gallisepticum* DNA, 0.25 μL Ex Taq (5U/μL) and 17.75 μL distilled water. Positive control (*M*. *gallisepticum* strain S6) and negative control (distilled water) were also performed concurrently for each reaction. The region of the gene *gyrA*, *gyrB*, *parC* and *parE* were amplified. Amplification was carried out at 94°C for 5 min, 94°C for 45 s, 55°C for 45 s, 72°C for 1 min for 30 cycles. The final extension cycles were at 72 ºC for 5 min.

### Analysis of quinolone efflux

In order to prove whether an active efflux mechanism was involved in danofloxacin resistance mutants, the mutants selected above for DNA extraction were also employed to determine the danofloxacin MICs in the presence of the two kinds of efflux pump inhibitors: carbonyl cyanide 3-chlorophenylhydrazone (CCCP) (20 mg/L) and reserpine (20 mg/L) using the broth dilution method as described previously [17, 26–28]. Danofloxacin was diluted in a two-fold series from 0.019–4.8 mg/L, and then the exponential-phase cells were added. CCCP and reserpine were individually added to a 1:100 dilution of cells. The plates were sealed with a gas permeable film and incubated in a 37°C, 5% CO_2_ humidified incubator.

## Results

### Susceptibility determination

The MIC, MIC_99_, and MPC of danofloxacin against *M*. *gallisepticum* strain S6 using the agar dilution method were 0.15, 0.1, and 2.4 mg/L, respectively.

### Effect of danofloxacin concentrations on the susceptibility changes

An HPLC method was developed to detect danofloxacin concentrations. The detection and quantification limit of HPLC-FD was 0.003 and 0.006 mg/L. The simulated C_0_ of danofloxacin in the *in vitro* model were 0.12, 0.38, 0.94, 1.74, 2.40, 8.31 and 13.55 mg/L. These values agreed very closely to the initial experimental design values of 0.1, 0.3, 0.675, 1.25, 2, 7, and 13 mg/L.

**Fig 2:**
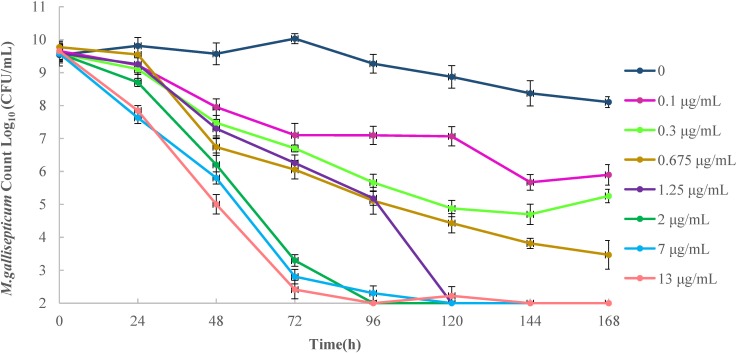
Effect of danofloxacin dose on inhibition of *M*. *gallisepticum* growth *in vitro*. The simulated C_0_ concentrations (where C_0_ is the initial danofloxacin concentration) were administrated once daily for 5 days. Colony-forming units were monitored at 24 h intervals before the initiation of danofloxacin in treatment and ending 2 days after the termination of danofloxacin treatment.

When the time course of danofloxacin killing was measured, the drug produced marked reductions in growth from 3.76 to 7.65 Log_10_ CFU/mL compared with the starting inoculum. The regimen in which the simulated C_0_ of danofloxacin (13 mg/L) in the *in vitro* model was achieved the maximal effect with a 7.65 Log_10_ CFU/mL reduction over 96 h. However, regrowth was observed in the regimen that simulated C_0_ values of of 0.1 and 0.3 mg/L in the *in vitro* model (Fig 2).

**Fig 3:**
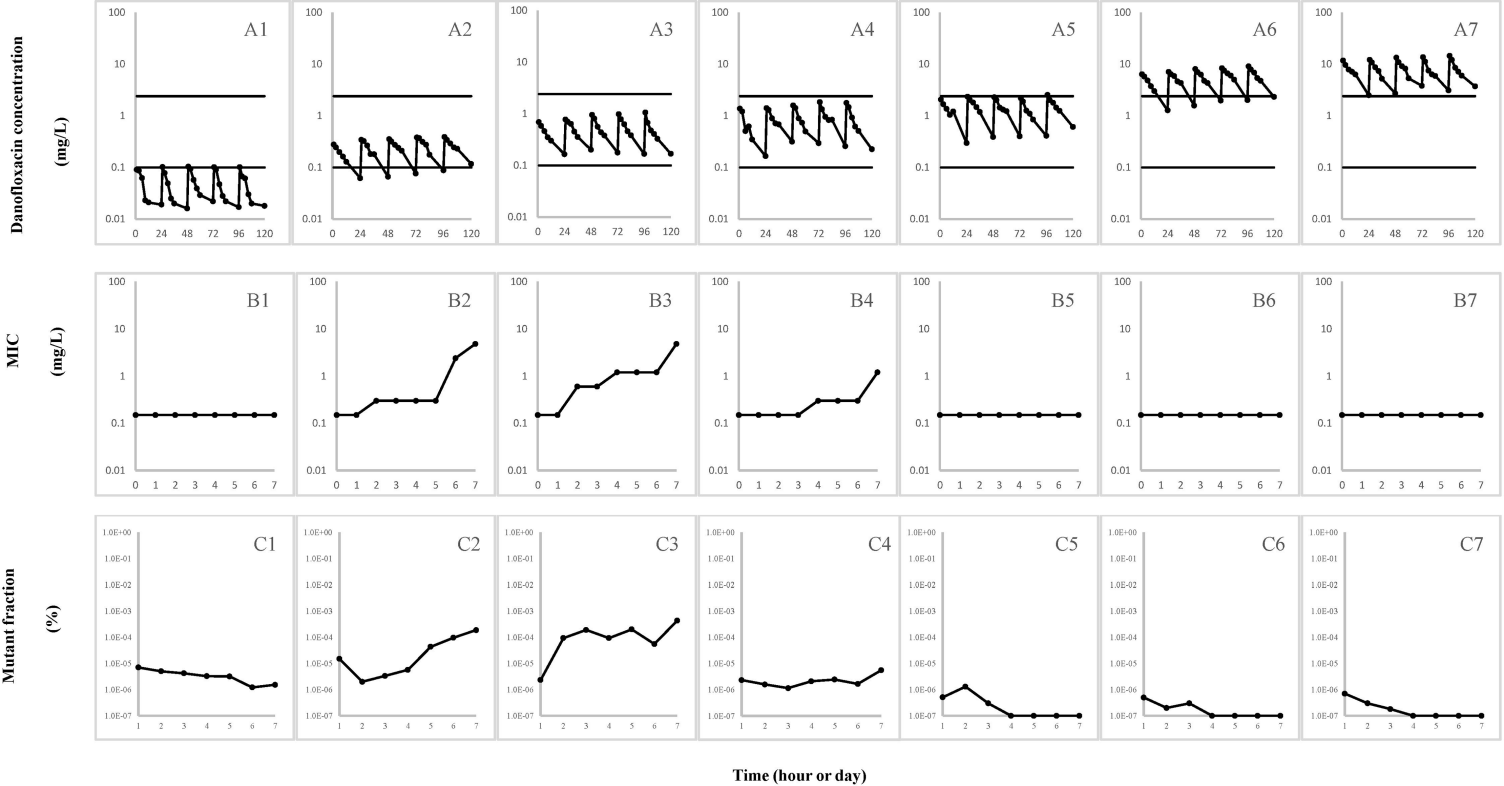
Effect of danofloxacin concentration on loss of susceptibility and mutant enrichment. *M*. *gallisepticum* strain S6 was inoculated into the IC and exposed for five consecutive days to once-daily danofloxacin replenishments in the *in vitro* dynamic model. Panels A1 to A7: Danofloxacin concentrations at the indicated times after the administration of each dose. Panels B1 to B7: Loss of susceptibility shown as an increase in average MIC for each group. Panels C1 to C7: The fraction of resistant mutants in each group was determined daily as the number of colonies grown on danofloxacin-containing agar (1 × MIC) relative to the number that grew on drug-free agar.

When *M*. *gallisepticum* was cultured at danofloxacin concentrations below the MIC_99_, at the upper part of the window, or above the MPC (Fig 3 panels A1, A5, A6 and A7), no resistant mutants with increased MICs were selected (Fig 3 panels B1 and C1; B5 and C5; B6 and A6; B7 and C7). When danofloxacin concentrations overlapped the lower window boundary or were at the lower part of the MSW (Fig 3, panel A2 and A3), the MIC increased prominently to 4.8 mg/L (Fig 3, panel B2 and B3). When danofloxacin concentrations at the middle part of the MSW (Fig 3, panel A4), this resulted in slightly decreased susceptibility because the MIC increased to 1.2 mg/L during danofloxacin treatment (Fig 3, panel B4). Therefore, selection of resistant mutants occurred when danofloxacin concentrations were in the lower portion of the selection window rather than the upper portion. This was indicated by increases in both the MIC and the fraction of mutants.

### Correlation of PK/PD Indices with mutant enrichment and amplification

We calculated the AUC_24h_/MIC_99_ and C_max_/MIC_99_ values to estimate the mutant enrichment of *M*. *gallisepticum* under danofloxacin treatment. Increase in the MIC did not occur when the C_max_ was below 0.1 or above 2 μg/mL. However, there was a loss of susceptibility under the simulated C_max_ concentrations of 0.3, 0.675, and 1.25 mg/L (Table 1). With the AUC_24h_/MIC_99_ and AUC_24 h_/MPC, the mutant selection window ranged from 9.04 h to 347.26 h and from 0.37 h to 14.47 h, respectively. When C_max_/MIC_99_ and C_max_/MPC were considered, the mutant selection window ranged from 1.15 to 24.03 and from 0.05 to 1, respectively (Table 1).

**Table 1:** Values of PK/PD parameters and change of MIC at different simulated concentrations.

The simulant C_0_ (mg/L)	AUC/MIC_99_ (h)	AUC/MPC (h)	C_max_/MIC_99_	C_max_/MPC	The increased MIC (mg/L)
0.1	9.04	0.37	1.15	0.05	no increase
0.3	62.91	2.62	3.77	0.16	4.8
0.675	113.37	4.72	9.42	0.39	4.8
1.25	179.17	7.47	17.45	0.73	1.2
2	347.26	14.47	24.03	1	no increase
7	1425.98	59.42	83.12	3.46	no increase
13	1938.74	80.78	135.47	5.64	no increase

### Mechanism of danofloxacin resistance in *M*. *gallisepticum*

There were no differences in the MICs and mutations before and after the passages. *M*. *gallisepticum* strain S6 and the four mutants with increased MIC values 0.3 (M1), 1.2 (M2), 2.4 (M3) and 4.8 (M4) mg/L were selected for QRDR gene analysis. The amino acid changes in *gyrA*, *gyrB*, *ParC*, and *ParE* of danofloxacin–resistant mutants are shown in Table 2. An amino acid substitution (Ser-83→Arg) was observed in the four mutant strains, corresponding to position 83 in the *gyrA* of *E*. *coli*. An additional amino acid substitution (Ala-64→Ser) was observed in M4, corresponding to position 64 in *parC* of *E*. *coli*. None of the four mutants possessed any base changes in *gyrB* and *parE*.

**Table 2:** Amino acid changes in *gyrA*, *gyrB*, *ParC*, and *ParE* in danofloxacin–resistant strains.

Strains	Mutations in QRDR target genes^a^:			
	*gyrA*	*gyrB*	*parC*	*parE*
S6	-	-	-	-
M1	Ser83→Arg	-	-	-
M2	Ser83→Arg	-	-	-
M3	Ser83→Arg	-	-	-
M4	Ser83→Arg	-	Ala64→Ser	-

-: No mutant was found.

^a^: *E*. *coli* numbering.

M1, M2, M3, M4: mutants are listed in order of increased MIC (agar dilution method) to 0.3, 1.2, 2.4 and 4.8 mg/L, respectively.

### Quinolone efflux mechanism

Four representative mutants (MI, M2, M3 and M4) were used to determine the MICs in the presence and absence of the efflux pump inhibitors CCCP and reserpine. Surprisingly, no MIC changes were found when CCCP and reserpine were added to the medium. This may prove that no active efflux mechanism was involved in danofloxacin resistance mutants.

## Discussion

In the past decades, the important place of the antibiotics that control bacterial infections has been threatened by the emergence and spread of antimicrobial resistance among nearly all pathogens [29]. The development of antimicrobial resistance in bacteria has been driven by sustained exposure to antimicrobials. The potential role responsible for antimicrobial resistance has been contributed by such selective pressure, which was accomplished by the long-time overuse and misuse of antibiotics [30]. *M*. *gallisepticum* is the major mycoplasma pathogen in poultry and quinolone resistance in *M*. *gallisepticum* has become common, hindering treatment and control efforts [31]. *M*. *gallisepticum* colonizes the respiratory tract of the infected chickens. The primary signs of *M*. *gallisepticum* infections include nasal discharge, keratoconjunctivitis, air sacculitis and depression [32]. The danofloxacin concentrations in lung tissues of the *M*. *gallisepticum* infected chickens were simulated by this *in vitro* model.

The PK of danofloxacin in *M*. *gallisepticum* infected chickens has been investigated by us in another study [24]. In the study, danofloxacin was orally administrated to the infected chickens once daily for 3 days by an established *in vivo M*. *gallisepticum* infection model. The concentrations of danofloxacin in lung tissues were analyzed. The results showed that the half-life (t_1/2_) of danofloxacin in lung was 9.2 h. Moreover, the PK parameters (C_max_) were dose dependent. A significant correlation (R^2^ = 0.995) was found between dose and C_max_ according to the linear relationship. If the C_max_ obtained from our current *in vitro* model (0.12, 0.38, 0.94, 1.74, 2.40, 8.31 and 13.55 mg/L) were used to calculate the dose in that *M*. *gallisepticum* infected *in vivo* model, the corresponding administration dose were 1.24, 2.14, 4.09, 6.87, 9.16, 29.67, 47.86 mg/kg, respectively.

In this *in vitro* model, maintaining danofloxacin concentrations above C_max_/MPC > 1 may be a straightforward way to restrict the acquisition of resistance. If C_max_/MPC > 1 in *in vitro* model were used to derive the dose in that *M*. *gallisepticum* infected *in vivo* model, the corresponding administration dose would be >9.16 mg/kg. Therefore, these findings suggest that danofloxacin may be effective to prevent the emergence of resistant mutants in *M*. *gallisepticum* infected chickens if administrated at a dosage above 9.16 mg/kg.

In the previous *in vivo* studies, increased efflux pump activity was displayed in the non-topoisomerase mutants that were selected from the lower and middle parts of the mutant selection window [33, 34]. Since many non-target resistance mutants will accumulate at low dosage, it is difficult to obtain DNA gyrase (target) mutants that selected from high dosage. However, in our study, concentration in the lower part of the window with low values of AUC/MPC resulted in a rapid enrichment and selection for a mutant subpopulation and target topoisomerase mutants. This difference was most likely the result of a number of factors: (1) the most susceptible and resistant cells were killed when the concentrations were in the upper portion of the window. This was caused by a greater MPC and MIC_99_ ratio (selection index) for danofloxacin; (2) A larger variety and abundance of preexisting resistant mutant subpopulations survived and propagated near the bottom of the window; (3) the efflux pumps were not activated.

The primary quinolone resistance mechanisms are mutations in DNA gyrase and topoisomerase IV, belong to the topoisomerase II family, which are significant for bacterial viability. Both enzymes are composed of two kinds of subunits. DNA gyrase is consisted of *gyrA* and *gyrB*; topoisomerase IV is consisted of *ParC* and *ParE* [35]. Our investigation revealed that all the mutants presented single mutation in *gyrA* and subsequent challenge added a *parC* mutation only in strain M4 with the highest MIC. This suggested that the MIC correlated with the number of mutations. The substitution of Ser83→Arg in the *gyrA* and the Ala64→Ser substitution in the *parC* had been previously described [36]. Unlike previous reports in which quinolone resistance in *M*. *gallisepticum* resulted from four different alterations in *gyrB*, we found no *gyrB* mutations in the mutant strains. Consistent with our results, the lack of *gyrB* mutations had been demonstrated with other mycoplasmas, such as *Ureaplasma urealyticum* and *Mycoplasma hominis* [37–39]. Stepwise resistance to danofloxacin was observed in our study when *M*. *gallisepticum* was sequentially challenged with increasing drug concentrations. This resistance mechanism was most likely similar to what occurs in Gram-negative organisms; DNA gyrase is usually the primary quinolone target and the *gyrA* mutation is the first common mutational step. Furthermore, resistance mutations in *parC* were established when DNA topoisomerase IV was subjected to higher drug concentrations [5].

To date, no other mechanism of quinolone resistance has been identified in *M*. *gallisepticum*. We expected that a secondary mutation would result in drug inactivation as described in other systems [40]. Indeed, energy-dependent efflux as a mechanism of quinolone resistance is found in Gram-negative and Gram-positive bacteria [41, 42]. We found no change of danofloxacin MICs in the presence of CCCP and reserpine against the danofloxacin susceptible strain (S6) and resistant strains (M1, M2, M3 and M4) that were selected from *in vitro* model. The ABC transporter (ATP-binding cassette) and the MFS transporter (major facilitator superfamily) are present in the *M*. *gallisepticum* genome, but they were most likely not acting on danofloxacin efflux. Danofloxacin is a substrate for multiple transporters, including P-gp (permeability-glycoprotein) and MRP2 (multi-drug resistance associated protein 2) that are both ABC transporters. The compounds can be pumped out of the the cellular cytoplasm by P-gp and MRP2. Additionally, danofloxacin (the transporter-dependent secretion of antimicrobial) can be transferred from the basolateral site to the alveolar space in bronchial and bronchiolar epithelium and endothelial cells. This would contribute a therapeutic advantage against *M*. *gallisepticum* if they colonize in bronchiolar and alveolar space [43, 44]. Hence, the danofloxacin concentrations in lung tissues are higher than in plasma in the tested animals including chicken, sheep and cattle [20, 45, 46].

The current study indicates that when drug concentrations fall inside the MSW, resistant mutants were selectively enriched. Maintaining drug concentrations above C_max_/MPC > 1 provides a framework for preventing the development of further resistance in *M*. *gallisepticum*. The single mutation in *gyrA* (Ser-83→Arg) was found in all mutants, while double mutations in *gyrA* and *parC* (Ala-64→Ser) were detected only in the mutant with the highest MIC. Although the abilities of danofloxacin to prevent the resistant mutant enrichment could be predicted by *in vitro* dynamic models, the MSW hypothesis also should be validated *in vivo*.

## Supporting information

S1 Table Nucleotide sequences of the primers used for PCR. (DOCX)

S2 Table Danofloxacin concentrations were monitored at the indicated times after the administration of each five dose in the *in vitro* model. (DOCX)
