# Supplementary material for: Relationship between danofloxacin PK/PD parameters and emergence and mechanism of resistance of Mycoplasma gallisepticum in In Vitro model
Source: PLoS One. 2018 Aug 29;13(8):e0202070. doi: 10.1371/journal.pone.0202070 (PMC6114503; doi:10.1371/journal.pone.0202070)
Supplement: S1 Table — (DOCX) [file pone.0202070.s001.docx]

**Supporting Information**

S1 Table. Nucleotide sequences of the primers used for PCR

| Primer | Sequence |
| --- | --- |
| *gyrA* | F 5’-TATGG TGCTTACACT TCAG-3’ |
|  | R 5’-CTACGGCAAT ACCACTTG-3’ |
| *gyrB* | F 5’-TGACGGTAAGATTAGCAAAG-3’ |
|  | R 5’-ACATCAGCATCGGTCATGA-3’ |
| *parC* | F 5’-ATGGATAAGAAAAAGGTATTTCAAAAAG-3’ |
|  | R5’-TTAACGAGTAAGTTAGGTAATAAACTAGGTAAGAT-3’ |
| *parE* | F 5’-GGTATCAAATTACAACGAAAAAC-3’ |
|  | R 5’-CCACCATCTTGGTAGATCGA-3’ |
