# Supplementary material for: Relationship between danofloxacin PK/PD parameters and emergence and mechanism of resistance of Mycoplasma gallisepticum in In Vitro model
Source: PLoS One. 2018 Aug 29;13(8):e0202070. doi: 10.1371/journal.pone.0202070 (PMC6114503; doi:10.1371/journal.pone.0202070)
Supplement: S2 Table — (DOCX) [file pone.0202070.s002.docx]

**Supporting Information**

S2 Table. Danofloxacin concentrations were monitored at the indicated times after the administration of each five dose in the *in vitro* model.

| Time (h) | The simulated C_0_ in the in vitro model (mg/L) | | | | | | | |
| --- | --- | --- | --- | --- | --- | --- | --- | --- |
|  | 0.1 | 0.3 | 0.675 | 1.25 | 2 | 7 | 13 |  |
| 1 | 0.09 | 0.28 | 0.69 | 1.37 | 2.05 | 6.35 | 11.7 |  |
| 3 | 0.09 | 0.24 | 0.58 | 1.21 | 1.67 | 5.71 | 9.6 |  |
| 6 | 0.06 | 0.20 | 0.46 | 0.50 | 1.36 | 4.82 | 7.8 |  |
| 9 | 0.02 | 0.16 | 0.35 | 0.62 | 1.04 | 3.73 | 7.1 |  |
| 12 | 0.02 | 0.13 | 0.30 | 0.35 | 1.21 | 3.01 | 6.3 |  |
| 24 | 0.02 | 0.06 | 0.16 | 0.17 | 0.30 | 1.27 | 2.5 |  |
| 25 | 0.10 | 0.34 | 0.77 | 1.41 | 2.33 | 7.03 | 12.1 |  |
| 27 | 0.08 | 0.32 | 0.70 | 1.30 | 2.02 | 6.36 | 10.7 |  |
| 30 | 0.05 | 0.26 | 0.64 | 0.89 | 1.79 | 5.85 | 8.6 |  |
| 33 | 0.03 | 0.18 | 0.45 | 0.71 | 1.47 | 4.61 | 7.3 |  |
| 36 | 0.02 | 0.18 | 0.35 | 0.68 | 1.18 | 4.31 | 5.2 |  |
| 48 | 0.02 | 0.07 | 0.20 | 0.31 | 0.39 | 1.57 | 2.7 |  |
| 49 | 0.10 | 0.35 | 0.95 | 1.58 | 2.29 | 8.01 | 13.3 |  |
| 51 | 0.09 | 0.32 | 0.81 | 1.42 | 2.01 | 7.01 | 10.9 |  |
| 54 | 0.06 | 0.27 | 0.56 | 0.89 | 1.44 | 6.26 | 9.1 |  |
| 57 | 0.04 | 0.24 | 0.44 | 0.73 | 1.31 | 4.74 | 8.2 |  |
| 60 | 0.03 | 0.21 | 0.38 | 0.50 | 1.22 | 4.29 | 5.3 |  |
| 72 | 0.02 | 0.08 | 0.18 | 0.29 | 0.40 | 1.96 | 3.8 |  |
| 73 | 0.10 | 0.38 | 0.98 | 1.84 | 2.18 | 8.31 | 13.6 |  |
| 75 | 0.09 | 0.37 | 0.78 | 1.33 | 1.87 | 7.46 | 11.1 |  |
| 78 | 0.05 | 0.31 | 0.62 | 0.95 | 1.25 | 6.59 | 7.5 |  |
| 81 | 0.03 | 0.28 | 0.46 | 0.83 | 1.09 | 5.97 | 6.3 |  |
| 84 | 0.02 | 0.18 | 0.38 | 0.84 | 0.84 | 4.98 | 5.9 |  |
| 96 | 0.02 | 0.09 | 0.17 | 0.26 | 0.41 | 2.01 | 3.1 |  |
| 97 | 0.10 | 0.39 | 1.06 | 1.77 | 2.53 | 9.03 | 14.4 |  |
| 99 | 0.07 | 0.34 | 0.67 | 1.47 | 2.01 | 7.89 | 12.1 |  |
| 102 | 0.06 | 0.29 | 0.47 | 0.92 | 1.79 | 6.82 | 8.5 |  |
| 105 | 0.03 | 0.24 | 0.41 | 0.62 | 1.43 | 5.33 | 7.1 |  |
| 108 | 0.02 | 0.23 | 0.33 | 0.51 | 1.25 | 4.74 | 6 |  |
| 120 | 0.02 | 0.12 | 0.17 | 0.22 | 0.61 | 2.31 | 3.7 |  |
